# Preliminary Study of a Modular MR-Compatible Robot for Image-Guided Insertion of Multiple Needles

**DOI:** 10.3389/fonc.2022.829369

**Published:** 2022-05-16

**Authors:** Amanda M. Aleong, Thomas Looi, Kevin Luo, Zhiling Zou, Adam Waspe, Satwinder Singh, James M. Drake, Robert A. Weersink

**Affiliations:** ^1^ The Institute of Biomedical Engineering, University of Toronto, Toronto, ON, Canada; ^2^ The Centre of Image Guided Innovation and Therapeutic Intervention in the Hospital for Sick Children, Toronto, ON, Canada; ^3^ The Department of Medical Biophysics, University of Toronto, Toronto, ON, Canada; ^4^ The Department of Radiation Oncology, University of Toronto, Toronto, ON, Canada

**Keywords:** robotics, needle insertion, MRI, brachytherapy, biopsy

## Abstract

Percutaneous needle-based interventions such as transperineal prostate brachytherapy require the accurate placement of multiple needles to treat cancerous lesions within the target organ. To guide needle placement, magnetic resonance imaging (MRI) offers excellent visualization of the target lesion without the need for ionizing radiation. To date, multi-needle insertion relies on a grid template, which limits the ability to steer individual needles. This work describes an MR-compatible robot designed for the sequential insertion of multiple non-parallel needles under MR guidance. The 6-DOF system is designed with an articulated arm to extend the reach of the robot. This strategy presents a novel approach enabling the robot to maneuver around existing needles while minimizing the footprint of the robot. Forward kinematics as well as optimization-based inverse kinematics are presented. The impact of the robot on image quality was tested for four sequences (T1w-TSE, T2w-TSE, THRIVE and EPI) on a 3T Philips Achieva system. Quantification of the signal-to-noise ratio showed a 46% signal loss in a gelatin phantom when the system was powered on but no further adverse effects when the robot was moving. Joint level testing showed a maximum error of 2.10 ± 0.72°s for revolute joints and 0.31 ± 0.60 mm for prismatic joints. The theoretical workspace spans the proposed clinical target surface of 10 x 10 cm. Lastly, the feasibility of multi-needle insertion was demonstrated with four needles inserted under real-time MR-guidance with no visible loss in image quality.

## 1 Introduction

The insertion of multiple needles for minimally invasive procedures such as prostate brachytherapy is a time-consuming task with needle deflection and tissue deformation presenting the primary challenges to accurate placement. In particular, the treatment of localized prostate cancer using focal therapies such as high-dose rate brachytherapy and thermal laser ablation presents an opportunity to optimize needle placement thereby minimizing the number of needles to be inserted and reducing the risk to the patient. To date, state-of-the-art magnetic resonance (MR)-guided needle insertion typically uses a template grid registered to the MR image space to place the needles at planned locations based on pre-insertion images (1). The patient must be moved out of the MR bore to place each needle and returned to the bore to acquire verification images, leading to a procedure time of 3-5 hours on average for a multi-needle case. Robotic systems are being explored to improve patient access in closed-bore MR systems and enable needle steering under image guidance. Eliminating the need to move the patient is expected to reduce the overall procedure time and improve the needle targeting accuracy. However, due to challenges associated with clinical translation, there has been a shift in focus from automated needle insertion under continuous MR-guidance to passive needle guidance with intermittent MR imaging for the verification of needle placement.

Existing robotic systems focus primarily on the guidance of single-needle procedures wherein each needle is removed before subsequent needles are inserted. The mechanics of inserting a single needle through soft tissue to reach deep-seated targets has been investigated extensively. Efforts to reduce needle deflection due to needle-tissue interaction has led to steering mechanisms that utilize axial rotation and lateral force at the base of the needle to adjust the needle trajectory (2–4).

A brief review of clinically-tested, MR-compatible robots provides insight into the limitations of available systems. For single needle insertion, the MrBot is a 6-DOF, pneumatically driven benchtop system developed at John Hopkins for use in the MR environment to facilitate transrectal prostate biopsy (5). The robot evolved out of a previously automated steerable system but was adapted for manual insertion to aid clinical translation. The FDA-approved system reported an MRI-based needle targeting accuracy of 2.55 mm (6). Few robotic systems have been developed for guiding multiple needle insertions. Recently, Cepek et al. presented MR PING, a 5-DOF bench-top guidance system currently undergoing clinical trials for focal laser ablation in the prostate (7). The system positions a small grid template *via* manual joint manipulation and is then locked in place once satisfactory alignment to the target has been achieved, with no subsequent steering possible once the grid is locked. Using the grid, needle insertions were confined to the same orientation resulting in a median needle guidance error of 3.5 mm over 37 insertions. Upon further inspection, needle deflection was identified as the main limiting factor in system accuracy. Another strategy for guiding multi-needle insertion was presented by Podder et al (8). The system was developed for ultrasound-guided brachytherapy seed placement. Simultaneous multi-needle insertion was achieved using actuated channels in a template grid to steer multiple needles. The results showed seed placement within 0.2 mm of the plan, confirming the benefits of needle steering. The systems presented limit the ability to steer non-parallel needles and constrains all needles to a single orientation thereby limiting access to optimal insertion paths for individual needles. The targeting errors observed for the first two systems presented are consistent with errors seen in the clinic for traditional template-based procedures.

In practice, the needle steering models that predict deflection based on tissue models and forces at the base of the needle are often complex and computationally expensive, limiting their feasibility in real-time applications (3, 9). Real-time MR offers additional feedback that may be used to reduce the model complexity and facilitate closed-loop control systems. As such, a robotic system that is compatible with real-time imaging is needed. Critically, there should be minimal impact on image quality when the robot’s joints are in motion. The signal-to-noise ratio (SNR) provides a suitable metric for gauging the impact of the robot on the MR. Furthermore, there is need for a system that overcomes the specific challenges associated with the insertion of multiple needles. Namely, entry-point and trajectory constraints imposed by placing needles next to each other and the need to change the needle that the robot is guiding without excessive movement of the patient. In addition, the presence of needles in the tissue affects the tissue deformation and target shift for subsequent needles.

This work presents a novel strategy to sequentially drive the insertion of multiple non-parallel needles under real-time MR-guidance. A modular robotic system is described with an articulated arm to extend the robot’s reach into a closed-bore MR system and a hinge-based needle guide to support needle release for subsequent needle insertions. This strategy enables the robot to maneuver around existing needles while minimizing the footprint of the robot. The system aligns the needle guide along a specified path and is designed to support future work on the adjustment of the needle trajectory to minimize needle deflection during insertion for each target point. This paper reports on the conceptual design and preliminary validation of a robot for sequential non-parallel needle insertion under continuous image guidance using custom pre-clinical gel phantoms designed to mimic the entry force properties of tissue. The gel phantoms allow for measurements in a controlled setting to confirm (and correct if necessary) the robot functionality before we move to further studies using *ex vivo* tissue samples and pre-clinical animal studies.

## 2 System Overview

### 2.1 Design and Specifications

The robot described herein is developed for transdermal needle insertion, in the inferior to superior direction, under real-time MR guidance, aligning with requirements described by recent guidance documents on image-guided robotic interventions (10, 11). Critically, the operation of the robot is designed to have no adverse impact on image quality by either its presence in the MR bore or during the robotic adjustment of a needle. All components of the robot are made with MR safe or MR conditionally safe materials according to American Society for Testing and Materials guidelines. The main components of the robot body were machined from Delrin, aluminum, brass, and acrylic. The end effector was 3D printed using stereolithography (PolyJet, Stratasys) using VeroWhite (Stratasys) plastic. Further, all parts were visibly inspected prior to assembly. The joints are actuated by non-magnetic ultrasonic rotary motors (USR30/60-E3M, Shinsei Co., Japan). The current system is built to accommodate passive, hand-driven insertion. The end effector i.e. the needle guide mechanism that attaches to the last link of the robot, is designed to be interchangeable to support future modifications for automation. For clinical use, the robot can be replaced with sterile covers and the 3D printed needle guide can be removed and sterilized using standard ethylene oxide gas protocols.

The planned robot workspace covers the full extent of the target organ (initially the prostate) with a constrained surface for needle insertion. It is designed to access all positions and orientations for needle entry-points for an expected range of patient sizes. Based on clinical experience for our initial scenario of prostate insertions, the target surface consists of a 10 x 10 cm transverse region allowing access to a 10 x 10 x 5 cm target cube up to 10 cm below the skin. The angle of approach covers a range between ±5° about the coronal and ±15° about the sagittal. The robot is also designed to accommodate access to the patient during needle insertion and allows easy release of the needle to facilitate multiple needle insertions. Both hardware and software safety interrupts are included to ensure patient safety. Furthermore, the motion of the robot joints are constrained to prevent collision with the patient, clinician, and magnet during normal operation.

Additional criteria considered when designing the robot are listed herein. The end effector should align to an entry point at the specified position and an orientation with a translational error under 1 mm and a rotational error under 1° in phantom tests to meet clinical requirements and remain competitive with existing systems (10–13). The system should be rigid enough to generate an insertion force of up to 2 N to allow the clinician to make adjustments at the needle entry point without causing unnecessary damage to the tissue or deformation of the robot links (14, 15). Furthermore, the motors and attached mechanisms should allow small adjustments to the needle trajectory for needle steering without interfering with other needles already in place. Specifically, adjustments on the order of 1 mm and 1° to the end effector position and orientation should be feasible without interfering with the clinician workspace.

To establish base functionality, the initial iteration of the system is built to accommodate hand-driven needle insertion *via* a custom needle guide while future modifications will support an automated approach.

### 2.2 Hardware

**Figure 1:**
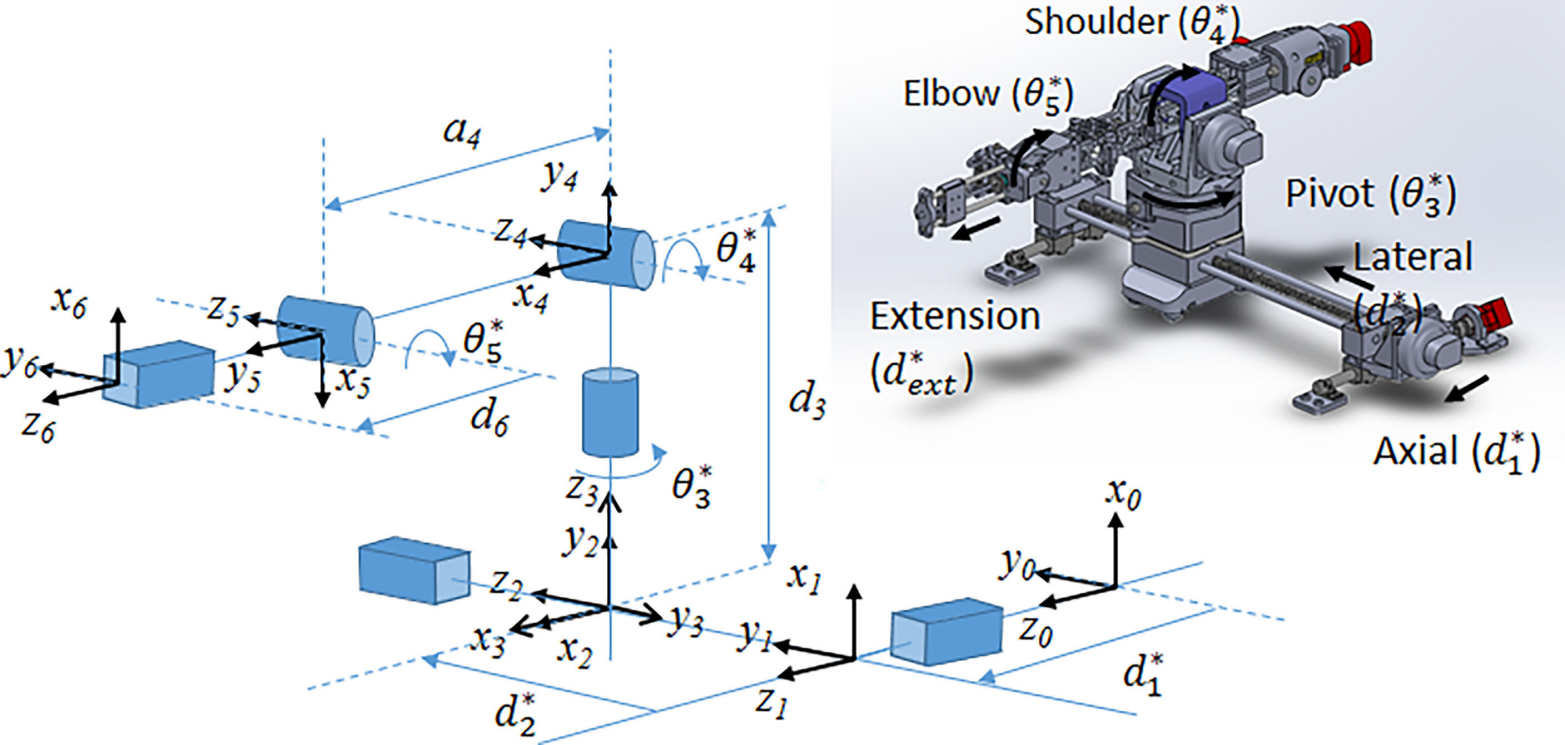
(Left) Joint-level schematic of robot showing coordinate systems and movement of each joint; (right) CAD rendering of the 6-DOF robot showing joint motion.

The system consists of the robot, the control box, and a computer console for interfacing with the robot and communicating with the MR. The robot consists of a planar Cartesian base and an articulated arm, able to move in 6 dimensions. It consists of 3 prismatic joints (P) and 3 rotary joints (R), as shown in **Figure 1**, with a PPRRRP configuration and an interchangeable end effector.

The 6-DOF robot is further divided into 3 sections. The base consists of two prismatic lead screw joints (axial and lateral) providing planar motion. The arm consists of 3 rotational joints: the pivot, the elbow and the shoulder. Lastly, the end effector attaches to a 1D lead screw for extension along the insertion direction. The elbow and end lead screw are driven by independent belt mechanisms.

**Figure 2:**
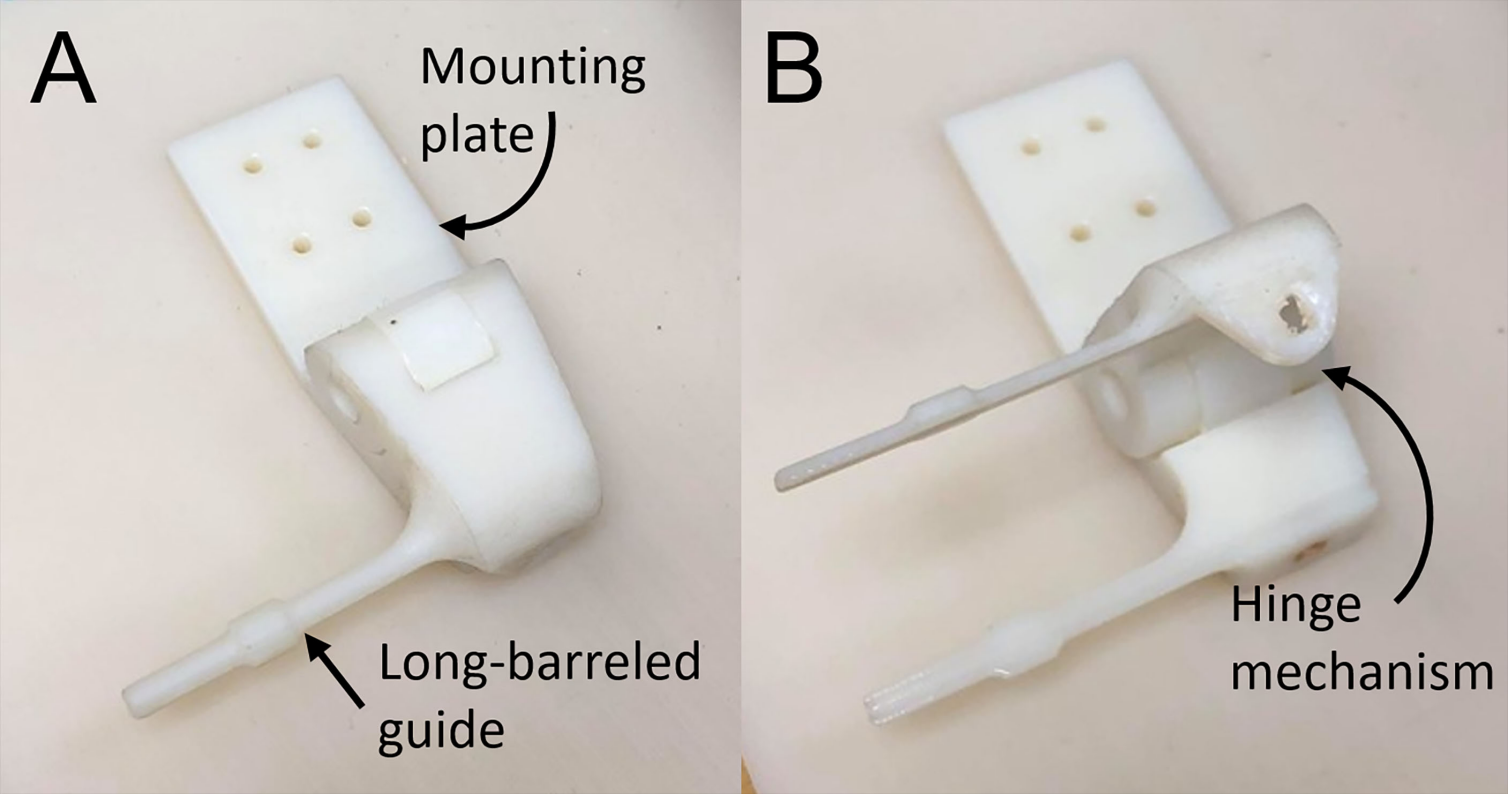
End effector needle guide in **(A)** closed and **(B)** open configuration.

The end effector for manual needle insertion, shown in **Figure 2**, was designed to enable needles to be easily swapped out on the robot, without undue effort on the clinician’s part or excessive movement of the patient bed. It enables the tip of the needle guide to be positioned as close to the skin as possible to minimize the deflection due to torque on the needle as it enters the tissue. To reduce the space between needles, the footprint of the needle guide was minimized to allow the sequential placement of multiple needles while still being rigid enough to guide the needle and maintain its trajectory. Lastly, the modular design of the robot end effector allows it to be easily swapped out or adapted for other modes of operation. The custom-designed 3D-printed needle guide is connected to the robot *via* a mounting plate and incorporates a hinge mechanism to release the needle after insertion. It features a long barrel to enable the needle guide to access the target surface while maneuvering around needles that have already been inserted.

The control box consists of 2 USB4 controllers (US Digital) and 7 motor drivers housed in a metal case with fans and vents placed for optimal cooling. The motor drivers were calibrated by Shinsei Co. for operation with 10 m MR-compatible shielded connecting cables. The control box connects to the robot *via* D-sub filtered connectors (API Technologies 56-705-003) which were used to minimize the electromagnetic impact of the robot on the MR field. Specifically, the connectors filter noise induced through communication between the control box outside the room and the robot inside the MR room.

### 2.3 Robot Control Workflow

**Figure 3:**
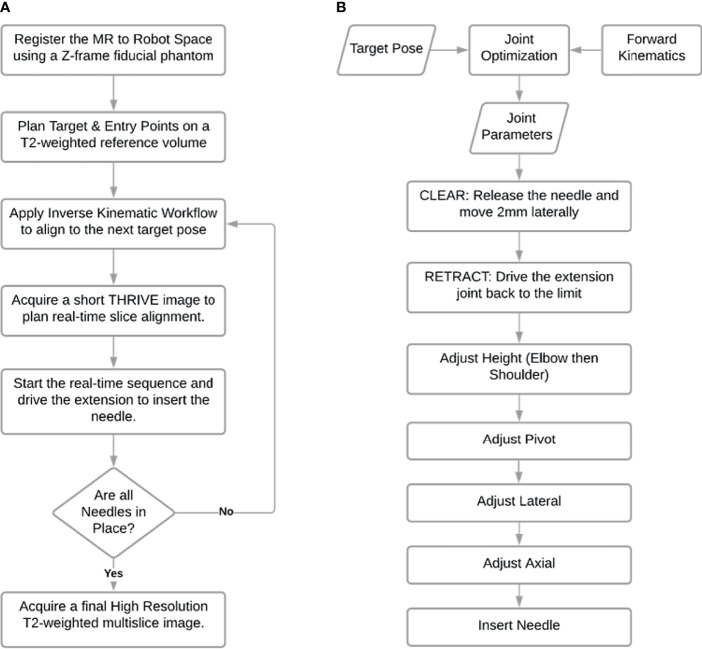
Workflow of needle insertions. **(A)** Procedure workflow; **(B)** Single needle insertion inverse kinematic workflow represented by third step in workflow **(A)**.

The robot control workflow describes the overall process from registering the robot with the MR-guidance system, to selecting the target, driving the robot and inserting a sequence of needles as shown in **Figure 3**. The robot is manually mounted on the MR bed and registered to the MR image space as described in 2.3.1. After registration, the Z-frame is removed by the operator and the target volume is imaged to select the desired needle poses. A plan is generated using the inverse kinematic workflow described in 2.3.3 to drive the robot to each target. With the target phantom still in the bore, the robot is driven automatically to the next target. The needle is then inserted into the guide and the operator has the choice to manually drive the needle insertion or automatically drive the needle in using the extension joint. At this point, the real-time MR slice is manually aligned to the expected insertion path and the sequence is initiated to monitor the needle during insertion. Once the needle is fully inserted further adjustments may be made by manual or automatic retraction of the needle. Automatic MR slice alignment and needle tracking is currently under development and is being reported elsewhere. Finally, the needle is manually released from the guide and the workflow proceeds to the next target. **Figure 3A** shows the procedure workflow and **Figure 3B** shows the inverse kinematic workflow for a single needle insertion as described in detail below.

#### 2.3.1 MR to Robot Registration

**Figure 4:**
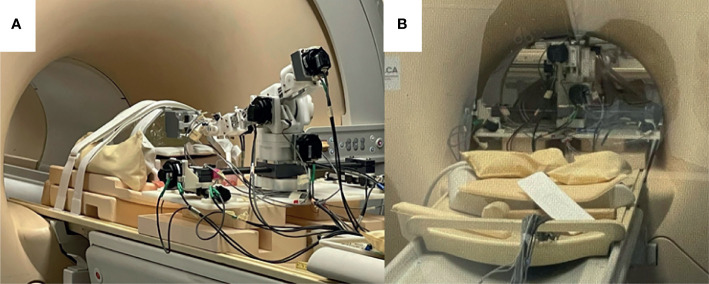
Robot setup in MRI bore. **(A)** Robot in place on MR bed with a z-frame fiducial phantom attached to the front of the extension joint; **(B)** Robot advanced into the bore of the MRI with phantom at isocenter.

A z-frame fiducial phantom was used to register the MRI coordinate space to the robot coordinate space (13, 16). The robot is placed on the MR bed and a homing system is used to drive the robot to a known joint configuration. The homing system consists of a set of limit switches used to detect when a joint has been driven to the edge of its working range. After driving each joint to their respective limit, the joints were then driven a known distance or angle to the desired home configuration and the Z-frame was connected rigidly to the last joint, as shown in **Figure 4**. By acquiring an image of the Z-frame, the pose of the Z-frame with respect to the magnet isocenter can be determined as described in (13, 16). Equation 1 describes the transformation of a point in MR coordinate space, **p** *_MR_*, to the same point described in the robot coordinate space, **p** *_Robot_*.


(1) $$p_{Robot}=\;T_{Z-Frame}^{Robot}*T_{MR}^{Z-frame}*p_{MR}$$

$T_{Z-Frame}^{Robot}$ is the transformation from the robot to the z_frame obtained by homing and $T_{MR}^{Z-frame}$ is the transformation from the Z-frame to the MR isocenter obtained by imaging the Z-frame. Using this mapping, a target selected in an MR image can be defined in robot coordinate space and passed to the inverse kinematic workflow to determine a suitable set of joint angles for needle alignment.

#### 2.3.2 Forward Kinematics

The pose of the end effector encompasses its position and orientation. In this section, we describe the forward kinematics formulation that maps the pose of each joint to that of the end effector. To begin, we define 6 joint variables (*d_1_, d_2_*, θ_3_, θ_4_, θ_5_, *d_6_*) each corresponding to a joint shown in **Figure 1**. The mathematical expression relating the inertial frame of reference, frame 0, to the end effector was then defined according to the modified Denavit-Hartenberg (D-H) convention (17). Using this convention, each joint is assigned a Cartesian coordinate frame and the following set of four D-H parameters may be used to determine the relationship between frame *i* and frame *i-1*. The link length, *a_i-1_*, describes the distance between **z** *_i-1_* to **z** *_i_* along the **x** *_i-1_* direction. The twist angle, α*_i-1_*, describes the angle from **z** *_i-1_* to **z** *_i_* about the **x** *_i-1_* axis. The joint offset, *d_i,_* describes the displacement between **x** *_i-1_* to **x** *_i_* along the **z** *_i_* direction. Lastly, the joint angle, θ*_i_*, describes the angle from **x** *_i-1_* to **x** *_i_* about the **z** *_i_* axis.

For the 5-DOF robot, 6 coordinate frames are required to establish the relationship between all the successive link-joint pairs, as shown in **Figure 1**. The transformation matrix, $T_{i}^{i-1}$ given by the expression in Equation 2 defines the translation and rotation of frame *i* with respect to frame *i-1* using the D-H parameters described for *i = 0, … , 5.*


(2) $$T_{i}^{i-1}=\;\left[\begin{array}{cccc} cos\theta_{i} & -sin\theta_{i} & 0 & a_{i-1}\\ sin\theta_{i}cos\alpha_{i-1} & cos\theta_{i}\text{sin}\alpha_{i-1} & -sin\alpha_{i-1} & -d_{i}sin\alpha_{i-1}\\ sin\theta_{i}sin\alpha_{i-1} & cos\theta_{i}sin\alpha_{i-1} & cos\alpha_{i-1} & d_{i}cos\alpha_{i-1}\\ 0 & 0 & 0 & 1 \end{array}\right]$$

**Table 1** summarizes the D-H parameters for the robot according to the modified D-H convention. With frames assigned to all the links, a series of matrix multiplications establishes the translation and orientation of frame 5 with respect to frame 0.


(3) $$T_{6}^{0}=\;T_{1}^{0}*T_{2}^{1}*T_{3}^{2}*T_{4}^{3}*T_{5}^{4}$$

**Table 1:** Joint definitions and D-H parameters.

Number (*i*)	Joint Type	Joint Name	Link Length *a*_(i-1)_	Twist Angle α _(i-1)_	Offset *d*_i_	Joint Angle θ_i_
1	Prismatic	Axial	0	0	*d_1_*	0
2	Prismatic	Lateral	0	-π/2	*d_2_*	-π/2
3	Rotary	Pivot	0	-π/2	*d_3_*	θ_3_
4	Rotary	Shoulder	0	π/2	0	θ_4_
5	Rotary	Elbow	*a*_4_	0	0	θ_5_ - π/2
6	Prismatic	Extension	0	-π/2	*d_6_*	π

Frame 6 is decoupled from the final pose of the end effector and is used primarily to advance the needle guide to the surface of the skin for hand driven procedures or ultimately to drive automated needle insertion along the intended direction. The final end effector offset assumes that joint 6 is fully extended.

The kinematic solution of the robot was determined by Equation 3. Using the D-H parameters from **Table 1** and the variable joint angles (*d_1_, d_2_*, θ_3_, θ_4_, θ_5_) the position and orientation of the end-effector can be computed. The final joint variable, *d_6,_* determines the proximity to the skin for the hand-driven case or the depth of insertion in the direction of joint 5 for the robot-driven case.

#### 2.3.3 Inverse Kinematic Workflow

**Figure 5:**
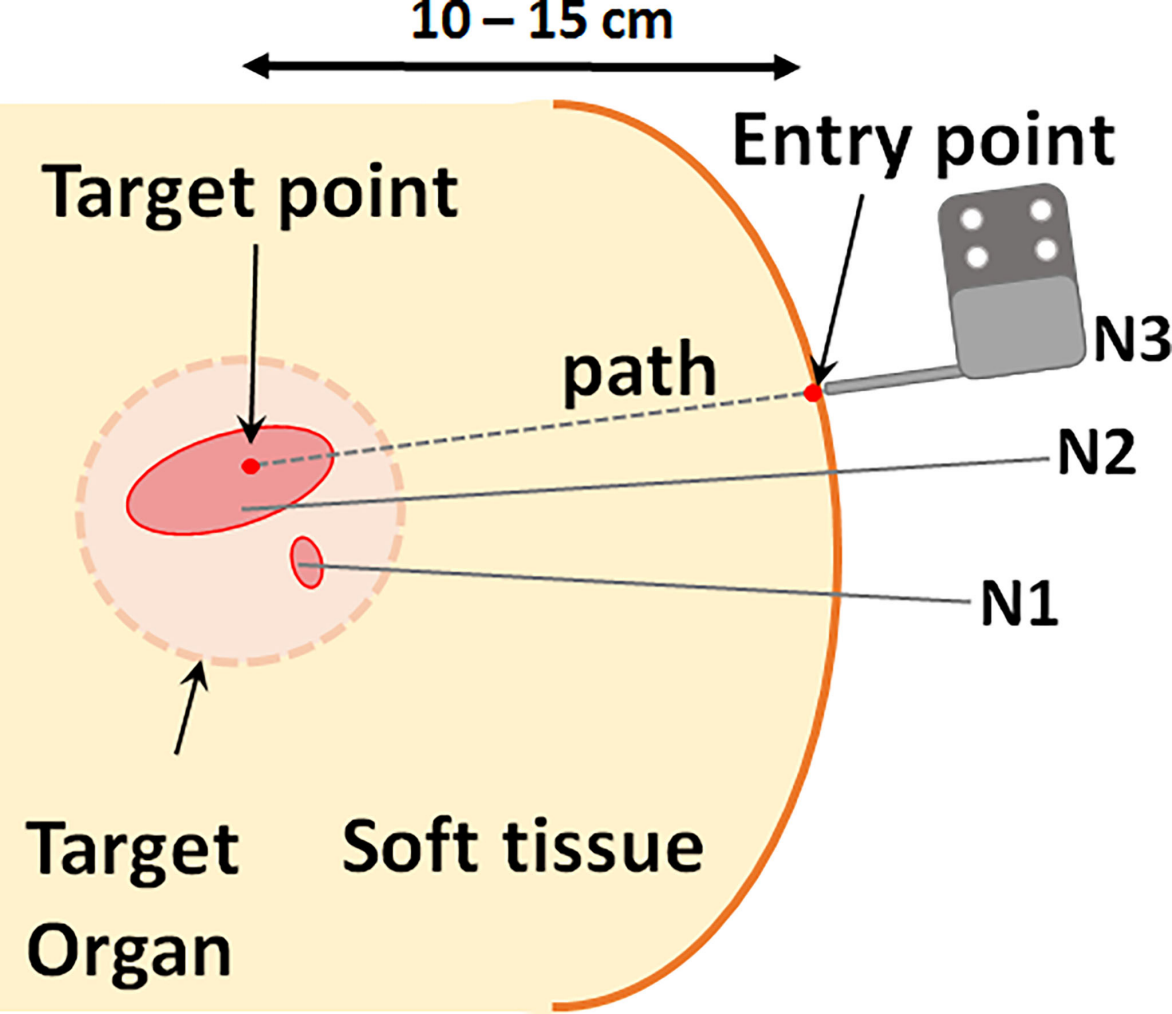
Schematic of needle typical clinical needle insertion scenario. Multiple needles are inserted at varying orientations for optimal coverage of the target volume. Each needle path, or pose, is defined by a point in the target and an entry point.

The inverse kinematics workflow described herein encompasses both the inverse kinematic formulation used to determine the specific joint configuration needed to place the end effector at a desired pose as well as the motion plan i.e. the sequence of joint motions needed to move to the target pose. To position the needle guide along the desired needle path, a pose is required based on an entry point through the skin and a target point inside the tissue (usually a tumor). Rather than being simply perpendicular to the axial plane, the orientation of the needle guide is usually defined by the need to have a treatment device align with the long axis of a target or to avoid intersecting other organs as shown in **Figure 5**. With the robot initially registered to a default home position, the joint configuration needed for alignment of the end effector to the target pathway is calculated by the joint optimization process described at the end of this section. As a critical step in sequential multi-needle insertion, the procedure to release the needle and clear its path is incorporated for all needles after the first insertion. In addition, the order of the joint motion is important due to patient proximity and is described in detail by the workflow shown in **Figure 3B**.

The inverse kinematics is formulated as a constrained optimization problem. It should be noted that the pivot directly determines the yaw angle while the shoulder and the elbow together determine both the height and the pitch angle of insertion, thus, accommodating for a range of entry points and patient sizes. The last joint (*d*_6_) acts as the insertion joint and therefore is not considered while computing the necessary joint configuration for a given target. The first five joint values are computed *via* inverse kinematics to position the last joint for insertion. In other words, the optimal joint configuration, as determined by inverse kinematics, positions the end of the last rotary joint at the desired needle insertion pose.

The inverse kinematics were computed in Matlab using the *fmincon* function, a non-linear solver for constrained optimization problems. Given a target pose, the solver uses a gradient descent algorithm to search the bounded joint space for a suitable set of parameters that minimize the Euclidean error between target position and the position of the end effector. The position of the end effector is computed by applying forward kinematics to each proposed set of joint values. The bounds of the search space are determined by the operating range for each joint. Further constraints are applied to define the desired orientation of the target. These are equality constraints,


(4) $$\theta_{3}=C_{1}.$$

(5) $$\theta_{4}+\theta_{5}=C_{2}.$$

where C_1_ and C_2_ are constants defined by the target yaw (the angle about the z-axis of frame 0) and pitch (the angle about the y-axis of frame 0) angles respectively. In summary, the solver tests a range of joint values within the bounded space and returns the joint values that minimize the error function.

## 3 Methods

### 3.1 MR Compatibility Test

The robot was tested on a 3T Philips Achieva system with a 60 cm diameter bore and, with minor modifications, will be compatible with similar clinical MRI systems. Four clinically-relevant image sequences were investigated for qualitative uniformity in the images as well as quantitative changes in SNR. T1-weighted turbo spin echo (TSE), T2-weighted TSE and ultrafast gradient echo (THRIVE) sequences may be used for anatomical landmarking and pre-operative treatment planning. High resolution T2-weighted TSE is additionally used to confirm the final placement of the needles in a typical brachytherapy procedure. The gradient echo-echo planar imaging sequence (FFE-EPI) provides a means to monitor needle insertion in near real-time using the magnitude portion of the image. The imaging parameters used for each of the sequences are summarized in **Table 2**. Two phantoms were used to assess the impact of the robot on the image quality for each type of sequence. A saline phantom was used to provide a standardized reference for quality assurance as is done at our institution. A gelatin phantom was used because it can be quickly and easily prepared and its similar water content to tissue results in MRI SNR and contrast that is generally representative of tissue. Further, their mechanical properties can be modified to mimic insertion into tissue. A saline phantom (Philips QA fluid grid phantom) was placed at the isocenter of the magnet and imaged with: (1) the robot in position ready for needle insertion but switched off (Power Off) and (2) the robot in place and powered on (Power On) and (3) the robot being remotely driven during acquisition (Moving). The process was repeated for a gelatin phantom. Additional scans were acquired in the gelatin phantom with a catheter and guiding titanium alloy trocar (6F) inserted (Trocar) while the robot was powered on. Finally, an image set was acquired with the guiding trocar removed and the catheter left in place (Catheter). Image signal-to-noise and qualitative uniformity were assessed for each image using the Philips DICOMViewer. The SNR was calculated as the difference between the mean signal in a region of interest (ROI) inside the phantom and the mean signal in a region outside the phantom divided by the standard deviation of the region outside of the phantom. Multiple ROIs (4-5 depending on the space) were used to obtain the average SNR across the phantom.

**Table 2:** Scan parameters for each MR sequence.

Sequence	Resolution (mm)	Slice Thickness (mm)	FA (°)	TE (ms)	TR (ms)
T2w TSE	1.56 x 1.56	3	160	79	4000
T1w TSE	1.56 x 1.56	3	160	10	750
THRIVE	1.56 x 1.56	1	15	2	20
FFE-EPI	1.56 x 1.56	5	19.5	20	25

### 3.2 Theoretical Workspace Simulation

The forward kinematics were used to simulate the workspace of the robot end effector in Matlab for the feasible range of joint angles. The workspace shows the volume of points that can be accessed by the end effector without considering the constraints imposed by the MR and patient which are subject to change. A sample segmentation of a prostate volume was obtained from a clinical case study and plotted as a reference landmark relative to the robot workspace. A potential needle trajectory is shown passing through the feasible workspace outside the skin and accessing the prostate which may be up to 10 cm superior of the skin.

### 3.3 Joint-Level Testing

The joint-level uncertainty was measured by repeatedly driving each joint over a set distance for a range of available joint values. Optical tracking (Polaris Vega, NDI, Waterloo, Canada) was used to assess the accuracy of each joint movement by fixing an optical marker to the end effector and recording its position and orientation in real-time. Each joint was driven back and forth, 10 times in each direction in steps of 5mm for prismatic joints and steps of 5° for revolute joints. Means and standard deviations were calculated for each isolated joint motion.

### 3.4 Workflow Assessment for Sequential Needle Insertion

To assess the feasibility of sequential needle insertion using the designed end effector, a benchtop test was performed in a gelatin phantom. A gel phantom was marked with seven insertion points on the proximal face of the phantom as illustrated in **Figure 9A**. Insertion points and insertion angles are reported as [*X, Y, R_x_, R_y_*], where *R_x_* and *R_y_* indicate rotation about the *X* and *Y* axis. Given the designed target surface of 10 x 10 cm, poses were selected at the upper and lower angled bounds of the target space (5: [50, 0, -15, 0], 3: [-50, 0, 15, 0]). Distances are in mm and angles are in degrees. Two poses were selected at the extreme left and right of the target space (1: [0, -50, 0, -10], 7: [0, 50, 0, 10]). Three parallel poses were selected in the central zone of the target surface to demonstrate the feasible resolution for horizontal and vertical insertion (4: [0, 0, 0, 0], 2: [0, 5, 0, 0], 6: [5, 0, 0, 0]). For this study a flat insertion surface was assumed with constant insertion depth (z-axis). The order of the targets was selected to follow a left to right insertion pattern, but future work will consider the optimal sequence for insertion to minimize target motion while avoiding collision with other needles. The robot was driven to each target position in the order shown in the diagram by entering the appropriate joint values with the needle guide closed. At each position, a needle was inserted *via* the needle guide at the desired pose. The needle was then released by opening the needle guide and the process was repeated for the other six entry points. Needle positions and orientations were chosen to demonstrate the range of motion of the robot.

### 3.5 Needle Insertion Under Real-Time MRI

The feasibility of sequential multi-needle insertion under real-time MRI was assessed in a gelatin phantom using the workflow described in 2.3. The robot was set up in the MR bore and registered to the imaging space as described in 2.3.1. A THRIVE image of the phantom was acquired using the imaging parameters from **Table 2**. Four target points were delineated at various positions and angles on the transverse surface of the phantom. For each target the inverse kinematics were determined using the bore-constrained robot range of motion. For each target, the needle guide was driven to the planned position and orientation and real-time MR images (FFE-EPI from Table II) were acquired while the needle was being inserted. To assess the image quality of the real-time sequence while a needle was being inserted, automated needle insertion was simulated by positioning the needle tip at the surface of the gel and taping the shaft of the needle to the fully retracted needle guide. The needle guide was driven forward, and the progression of the needle was visualized under continuous FFE-EPI. After all the needles were inserted, a final T2w-TSE was acquired with trocars removed to assess the placement of the needles.

## 4 Results

### 4.1 MR Compatibility

**Figure 6:**
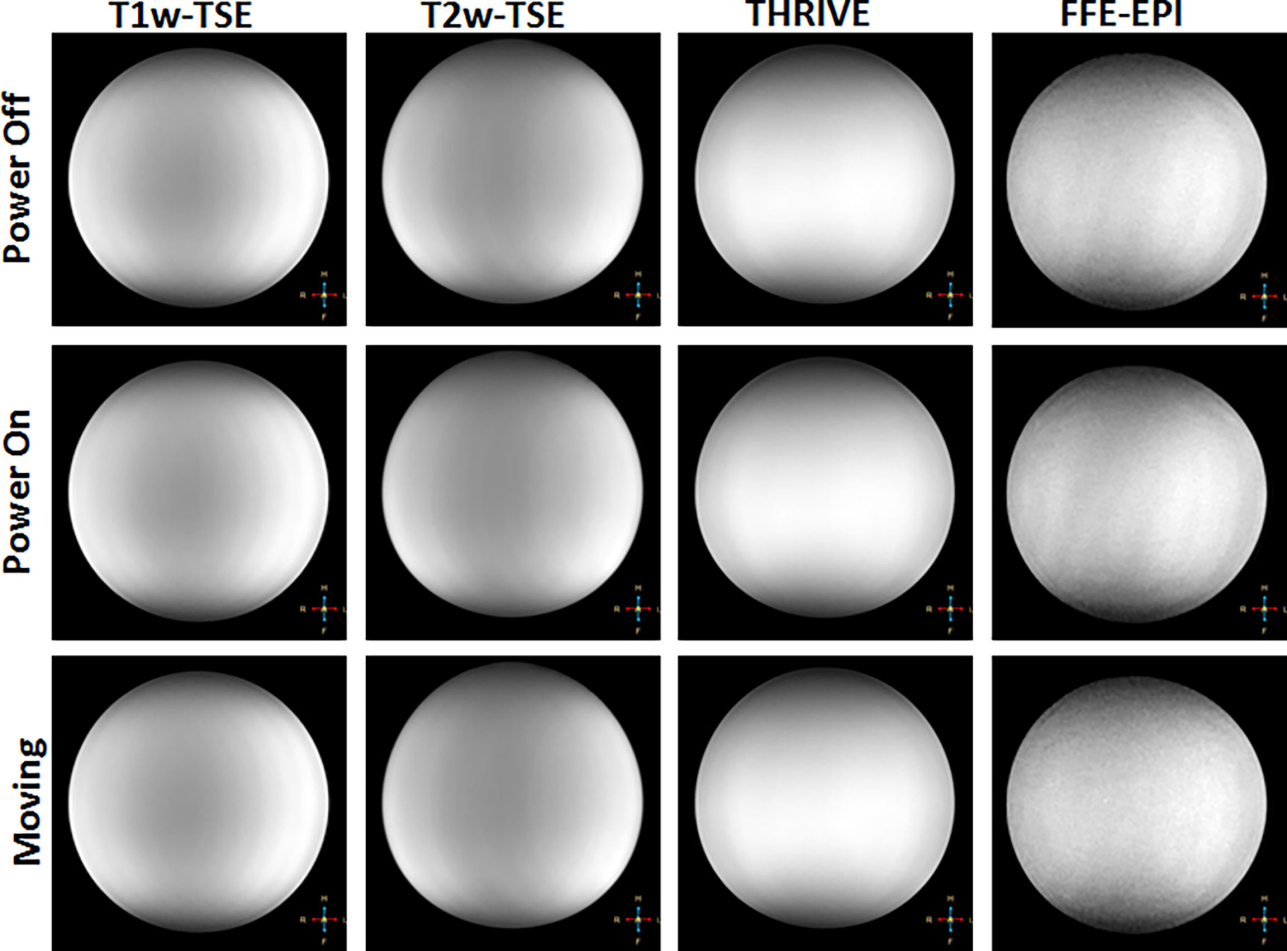
Representative images showing qualitative changes in MR images of a saline phantom for different sequences (in columns) and with the robot powered off (top row), powered on (middle row) and robot moving (bottom row).

**Figure 7:**
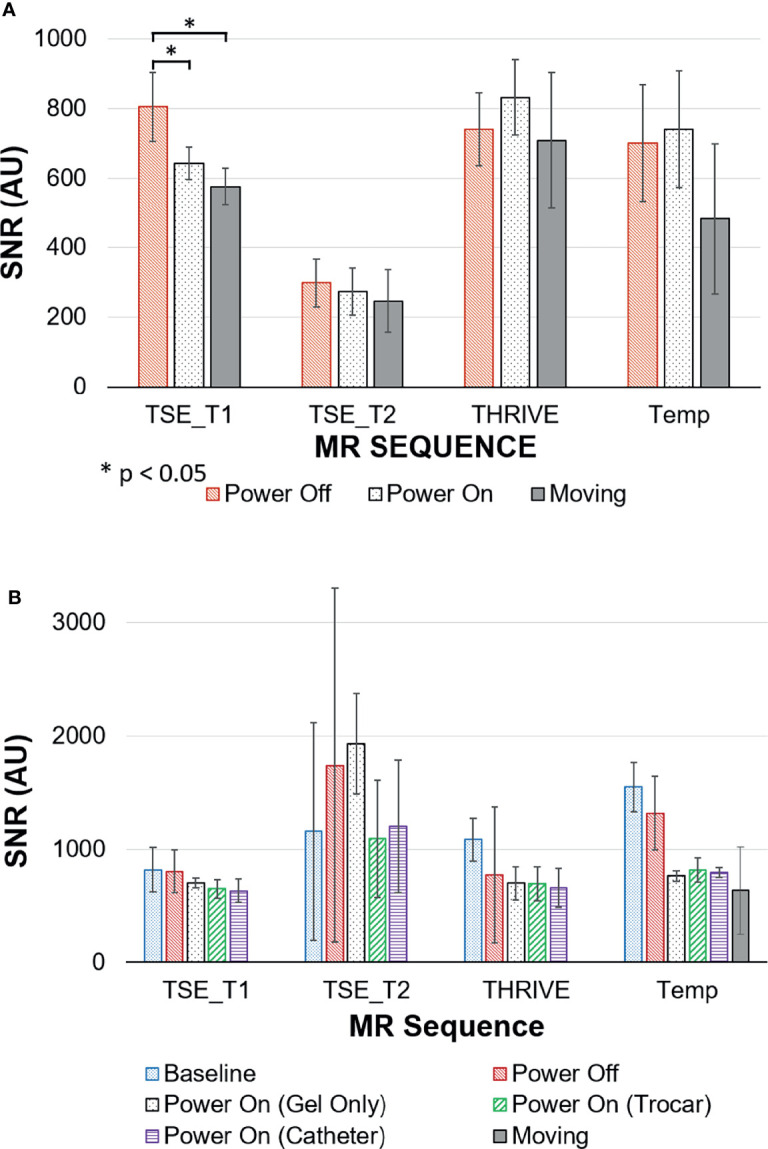
Signal to Noise ratios (SNR) for MRI sequences with the robot in different states of use measured in **(A)** saline phantom; **(B)** gelatin phantom.

**Figure 6** shows representative images of the Saline phantom for each sequence and robot condition. No visible change is observed when the robot is powered on or moving thus confirming the qualitative uniformity of the images for the working robot. The measured SNR values for both the saline and gelatin phantom are summarized by the graphs in **Figure 7**. The graph shows the mean over the SNR samples and the standard deviation represented as error bars. The four to five SNR samples measured for each case was used to run a one-way analysis of variance test to determine whether there was a significant difference in the group means for each type of sequence. The most dramatic change in SNR was observed by powering on the robot. In the saline phantom, a significant difference ( p < 0.05) was observed for the T1-weighted TSE (p = 0.0083). Using a multiple comparison test, it was determined that power off was significantly different from power on (p = 0.0467) and moving (p = 0.0076). In the gelatin, the SNR showed greater variability for each case compared to the saline phantom and no significant difference was observed for the group means for each sequence.

### 4.2 Theoretical Workspace

**Figure 8:**
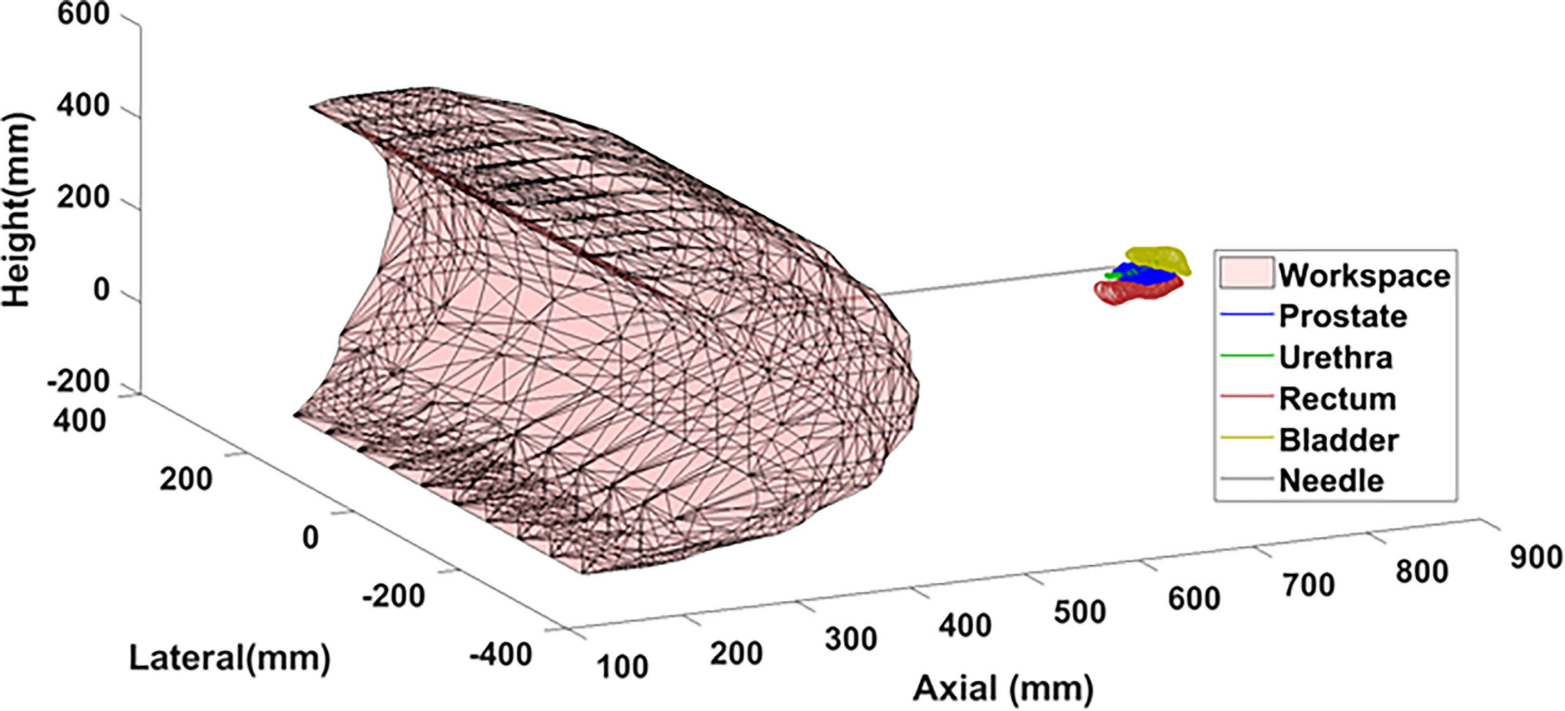
Theoretical workspace computed using forward kinematics relative to sample anatomical landmarks.

The robot spans 65 cm in height and 30 cm laterally with 6 cm in depth at its widest point. The workspace covers the proposed clinical target surface of 10 x 10 cm. This is confirmed by the plot of the feasible workspace of the robot relative to a sample anatomical target (**Figure 8**).

### 4.3 Joint Level Accuracy

The mean error and standard deviation for each joint are reported in **Table 3**. Joint level testing showed errors between (0.08 ± 0.05) to (0.31 ± 0.60) mm for the lateral and axial prismatic joints. The revolute joints powered by belt-drive resulted in the largest errors of (2.10 ± 0.72) to (1.80 ± 0.48) ° for the shoulder and elbow respectively. The majority of the joint inaccuracy for the revolute joints appears to be systematic and the precision of the joint motion falls within the design specifications of 1 mm for prismatic joints and 1° for revolute joints.

**Table 3:** Measured joint level errors.

	Error Mean	Error Standard Deviation
Axial (mm)	0.31	0.60
Lateral (mm)	0.08	0.05
Pivot (°)	0.20	0.19
Shoulder (°)	2.10	0.72
Elbow (°)	1.80	0.48

### 4.4 Workflow Assessment for Sequential Needle Insertion

**Figure 9:**
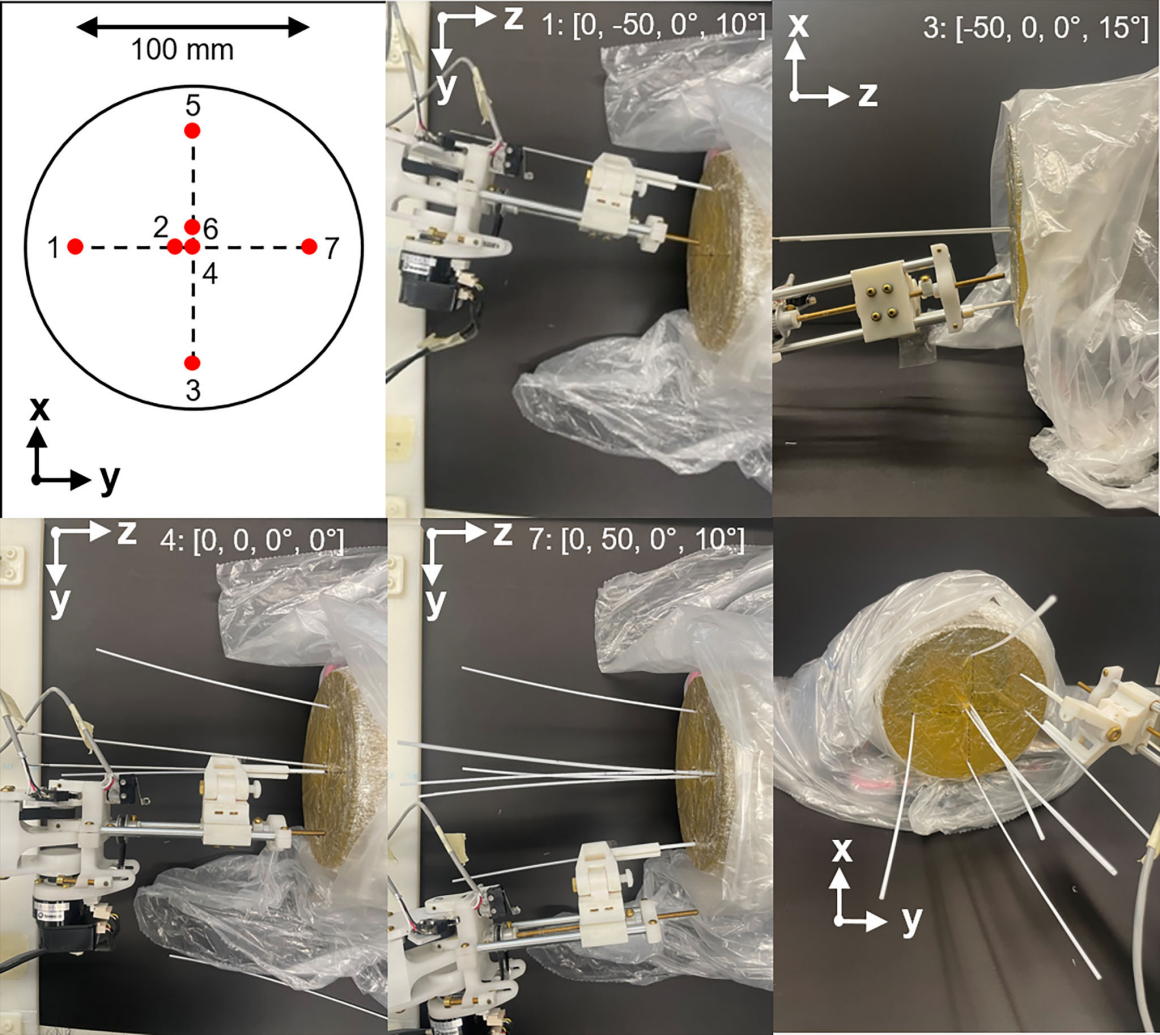
Example of multiple needle insertion by the robot using a gel phantom. Top left: needle insertion plan with numbers indicating order of insertion. In order from top-middle to bottom middle: photographs showing needle insertions at needles 1, 3, 4 and 7. Axis indicators show the perspective of each photograph. Insertion points and insertion angles are shown in each photograph as [*X, Y, R_x_, R_y_*]. Bottom right: isometric view after insertion of all needles.

**Figure 9** shows photographs of the needle guide and phantom during needle insertion. In order from top-middle to bottom middle: photographs after insertions of needles 1, 3, 4 and 7. Each needle was successfully placed at its respective target point without interference with other needles, demonstrating the maneuverability of the robot for multi-needle insertion. Needles were placed at four boundary points of the desired target surface to confirm the range of motion of the robot. In addition, three needles were placed in the central zone of the phantom with 5mm spacing in the horizontal and vertical directions to demonstrate that the feasible resolution of needle placement is consistent with that of a standard clinical grid template. **Figure 9** (bottom right) shows the final arrangement of the needles in an isometric view with the needle guide in place just after release of the last needle. The offset between needles 2 and 4 was 5 mm, well within clinical requirements.

### 4.5 Needle Insertion Under Real-Time MRI

**Figure 10:**
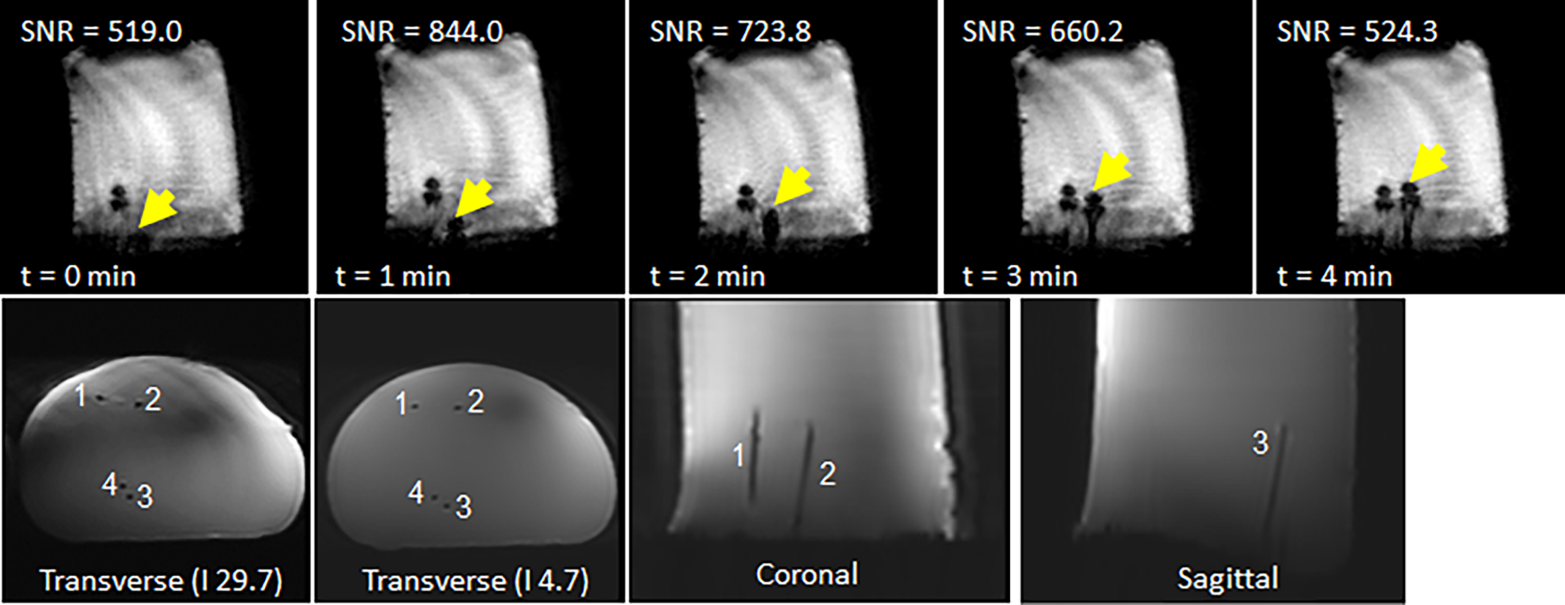
(top row) Representative time-lapse images of showing progression of a second needle inserted to the right of a needle already in place. (bottom row) Final scan of phantom after insertion of four needles: (left) axial scan close to phantom surface; (middle) axial scan close to needle tips; and (right) coronal scan.

The four needles were successfully inserted using the robot under real-time imaging. **Figure 10** shows the time-lapse images of a second needle being inserted to the right of the first needle. A video of the needle insertion is available in the supplementary material. The final TSE image of the phantom shows the four needles in place at various orientations with a 49 mm spread in the anterior-posterior direction and lateral spread of 22 mm.

## 5 Discussion

The results demonstrate the feasibility of robotically-guided multi-needle insertion under real-time MR guidance tissue-simulating gel phantoms. The robot showed minimal effect on the quality of the MR images and, crucially, sufficient signal was retained to visualize the needle during robot-driven insertion under real-time MR. Our plans are to progress to *ex vivo* and *in vivo* pre-clinical samples to verify these results in scenarios more representative of the clinical setting.

MRI is routinely used for guiding therapies because its soft-tissue contrast enables identification of targets, such as in the prostate, gynecology and neurological tumors. For prostate focal therapy, treatments such as high dose rate brachytherapy and phototherapies require insertion of multiple needles into the target. Compared to other MR-guided robotic systems, which insert parallel needles, our system is capable of inserting multiple needles at different orientations. We demonstrated that multiple needles could be inserted independently in both horizontal and vertical planes without interference from neighboring needles, with a minimum separation of ~5mm. We don’t anticipate any limit in the number of needles that can be inserted except in the space between and the orientations of the inserted needles. For prostate focal therapies, the typical number of catheters ranges between 3-10 needles, similar to our demonstration of seven needles. We therefore expect that our robot can be applied to these treatments. By inserting multiple needles at different orientations, we anticipate that better targeting can be achieved with fewer needles. Each needle can be appropriately angled to fully cover the target while avoiding other structures that could interfere with insertion, such as the pubic arch for lateral tumors, the urethra for medial and anterior targets, or the rectum for posterior targets. With fewer catheter insertions, a reduction in total procedure time may also be observed.

The SNR analysis revealed that the robot did not have a significant impact on the signal in the case of the tissue-like gelatin. Large fluctuations were seen in the gelatin. In all cases low values were observed for the background signal with values ranging from 0.48 to 10.13. As such, small changes in the noise had a severe scaling effect on the estimation of the SNR. This suggests that fluctuations in the noise of the image was a major contributing factor to the wide spread seen for the SNRs. Another contributing factor may be local inhomogeneities in the gelatin phantom. In contrast, the standardized saline phantom images showed relatively stable SNR values and provided a baseline reference supporting the hypothesis that the robot had no major impact on the signal quality with the exception of the T1-weighted TSE. Despite the change in SNR, it was determined that sufficient signal was retained to ensure the viability of the robot as an insertion tool in the MR environment.

Joint level testing confirmed that the robot will conform to the desired specifications with a few modifications required. Currently, the elbow joint is powered by a drive belt through a worm gear to the ultrasonic motor. During joint testing, the elbow exhibited a form of hysteresis whereby driving the joint upwards (against gravity) was slightly different versus driving downwards (towards gravity). Further inspection points to a possible improvement as the height of the belt teeth is too short resulting in the belt not engaging with the gear connected to the joint. The largest errors were observed due to backlash when changing direction. This may be due to both the spacing of the belt teeth and slack in the belt tension when engaging the driving gear in the opposite direction. In the joint test data, similar behaviour was observed for the shoulder joint where the inner gear of the gearbox may be slipping. Another possible source of error could be the plastic worm gears used in the pivot and elbow joints which can exhibit some deformation at the gear teeth. For future work, these plastic worm gears will be replaced with aluminum-based worm gears.

The preliminary workflow test for multi-needle insertion revealed several important factors for future consideration. The end effector requires construction from a stronger material with greater precision to minimize any slack in the needle guide during insertion. The order of insertion must be defined during the planning phase to ensure minimal interference between needles during insertion. Future work will explore the automatic optimization of the needle insertion plan and establish communication between the planning software and the robot control system. The current setup requires manual release of the needle which is not ideal for an in-bore patient set-up. A major benefit of the current design is the ease with which new end effectors each may be adapted to a specific needle type or procedure. Examples include angling the body of the needle guide for ease of access, changes for left or right-handed insertion and changing the gauge of the guide hole. To account for the characterized joint inaccuracies, an offset was applied during the MR guidance test.

Further evaluation of the functional workspace, the targeting accuracy, and the force output of the system is in progress. Additional tests will be conducted to assess the cumulative error associated with driving the robot in free space with and without the presence of a magnetic field. Lastly, the end effector will be modified to achieve automated needle insertion. 

The large needle artefact observed in the MR images is a characteristic of imaging at 3 Tesla. It is expected that for a typical 1.5T clinical scanner the needle localization will improve. With real-time MR images of the needle being fed back from the scanner, an automatic AI-based needle segmentation may be used to localize the needle in the image and predict deviations from the target path. We hypothesize that using such an approach will aid in earlier detection of needle deflection and will allow smaller adjustments at the entry point to correct for needle deflection, compared to intermittent verification images. To support small adjustments, a secondary mode of operation may be employed by the robot. In the case of needle correction, the kinematic workflow must be modified as the needle is already partially inserted in the patient, generally along the planned path but with some deviation of the tip from the planned trajectory. As such, the motion along several coordinates is constrained by tissue. The user will have the ability to switch between multiple control pathways to minimize needle deflection and overcome the tissue restrictions. For example, the user may employ the following strategy: (i) retract along extension until the needle is behind the point of deflection and (ii) adjust the joint configuration so that position of the end effector tip is maintained but is in a slightly different orientation. The optimal strategy for correction of needle deflection requires further investigation but will benefit greatly from the information on the true needle position provided by real-time MRI.

## 6 Conclusion

The robot described in this paper supports multi-needle insertion under real-time MR-guidance. The robot is specifically designed to allow individual needle manipulation and thus enables future implementation of corrective motions to minimize needle deflection during insertion. The robotic system, equipped with real-time MRI feedback, has the potential to improve the safety and efficiency of MR-guided percutaneous procedures such as prostate brachytherapy.

## Data Availability Statement

The raw data supporting the conclusions of this article will be made available by the authors, without undue reservation.

## Author Contributions

AA conceptualized and assisted in the robot design, assisted in the kinematics design, developed registration with MR imaging and tested its performance and wrote the manuscript. TL conceptualized, designed and assembled equipment and assisted in testing and assisted in writing. SS designed kinematics. KL designed and assembled equipment. AW assisted with MRI design and imaging. ZZ developed user interface and robotic homing system. JD provided resource towards the robot design and testing facilities. RW conceptualized and assisted in the robot design and co-wrote the manuscript. All authors contributed to the article and approved the submitted version.

## Funding

This research was supported by Siemens Healthcare Canada Ltd., Canadian Institutes of Health Research Mitacs Accelerate Program (Grant No MITACS IT12238) and the Natural Sciences and Engineering Research Council (Grant No 506721). Siemens Healthcare Canada provided direct funding to the project through the sponsored research agreement between Siemens Healthcare Canada and RW (through University Health Network, Toronto, CA, UHN-2015-MR-1-Jaffray) and by matching the contribution of Mitacs Accelerate Program in support of the graduate student. They had no influence on the experiments or the content of the manuscript and have no exclusive rights to the results or output of the research.

## Conflict of Interest

AA received support from Mitacs Accelerate Program.

The remaining authors declare that the research was conducted in the absence of any commercial or financial relationships that could be construed as a potential conflict of interest.

This research was supported by Siemens Healthcare Canada Ltd., and the Natural Sciences and Engineering Research Council.

## Publisher’s Note

All claims expressed in this article are solely those of the authors and do not necessarily represent those of their affiliated organizations, or those of the publisher, the editors and the reviewers. Any product that may be evaluated in this article, or claim that may be made by its manufacturer, is not guaranteed or endorsed by the publisher.

## Supplementary material

The Supplementary Material for this article can be found online at:: https://www.frontiersin.org/articles/10.3389/fonc.2022.829369/full#supplementary-material
